# Taurine Induces an Ordered but Functionally Inactive Conformation in Intrinsically Disordered Casein Proteins

**DOI:** 10.1038/s41598-020-60430-7

**Published:** 2020-02-26

**Authors:** Mohd Younus Bhat, Laishram Rajendrakumar Singh, Tanveer Ali Dar

**Affiliations:** 1 Department of Clinical Biochemistry, University of Kashmir, Srinagar, J&K 190006 India; 2 Dr.B. R. Ambedkar Center for Biomedical Research, University of Delhi, Delhi, 110007 India

**Keywords:** Protein aggregation, Protein aggregation, Chaperones, Chaperones, Intrinsically disordered proteins

## Abstract

Intrinsically disordered proteins (IDPs) are involved in various important biological processes, such as cell signalling, transcription, translation, cell division regulation etc. Many IDPs need to maintain their disordered conformation for proper function. Osmolytes, natural organic compounds responsible for maintaining osmoregulation, have been believed to regulate the functional activity of macromolecules including globular proteins and IDPs due to their ability of modulating the macromolecular structure, conformational stability, and functional integrity. In the present study, we have investigated the effect of all classes of osmolytes on two model IDPs, α- and β-casein. It was observed that osmolytes can serve either as folding inducers or folding evaders. Folding evaders, in general, do not induce IDP folding and therefore had no significant effect on structural and functional integrity of IDPs. On the other hand, osmolytes taurine and TMAO serve as folding inducers by promoting structural collapse of IDPs that eventually leads to altered structural and functional integrity of IDPs. This study sheds light on the osmolyte-induced regulation of IDPs and their possible role in various disease pathologies.

## Introduction

A well-defined, stable three dimensional structure was once thought to be an essential pre-requisite for proper function of a protein^1–3^. However, during the last few decades biologically active proteins without well-defined stable tertiary structures have also been found in most of the proteomes^4–6^. These proteins are often referred as intrinsically disordered proteins (IDPs) or natively unfolded proteins^7–10^. One of the distinctive feature of IDPs is the presence of relatively low number of hydrophobic and aromatic amino acid residues and high number of charged and polar residues^11–15^. The absence of stable structure allows these proteins to exist as highly dynamic conformational ensembles containing a wide variety of rapidly interconverting structures, thereby providing means for interaction with multiple often unrelated partners and for performing various important physiological functions^12,15–19^. These proteins once known as a small group of rare exceptions are being presently explored for their central roles in the regulation of various key cellular processes including signal transduction and molecular recognition, mRNA metabolism, translation, transcription, and cell cycle^8,10,15,18,20,21^. Additionally, many of such IDPs function as chaperones in different tissues^22^. More importantly, numerous IDPs have been found to be associated with the pathophysiology of various human diseases including diabetes, amyloidosis, neurodegeneration, cancer and cardiovascular diseases etc^9,19,23–25^.

The general mechanisms for the functional regulation of IDPs have been enigmatic. It has been known that the degree of disorderness is one signifying feature for the appropriate functioning of the IDPs^9,17,21,26,27^. A flexible structural organization helps various IDPs to interact with multiple binding partners so as to gain multiple functions^9,16,19,28^. Furthermore, alternative splicing affects intrinsically disordered polypeptide segments and thereby controls their regulatory and signalling functions^10,29–31^. Various post translational modifications also help to regulate numerous functions of IDPs^8,10,32^. For instance, methylases, kinases, and acetylases (as well as many other enzymes catalysing posttranslational modifications of proteins) may result in different signalling outcomes in various IDPs. One of the most important posttranslational modification, phosphorylation, plays a significant role in modulating conformational ensemble of IDPs owing to the presence of predominant sites for phosphorylation^5,10,33^. In addition to these regulatory mechanisms, recent studies have shown that small molecule co-solutes known as osmolytes, generally accumulated by cells under stress conditions, also have potential to regulate the functioning of IDPs and thus could modulate the regulatory network of cells^9,34,35^. These osmolytes have been grouped into three main classes: polyols (glycerol and sorbitol) and sugars (glucose and sucrose); amino acids (glycine and proline) and their derivatives (β-alanine and taurine), and methylamines (trimethylamine N-oxide (TMAO), sarcosine and betaine)^9,35–37^. In the present study, we have investigated the effect of osmolytes, belonging to different classes, on the structural and functional properties of two illustrative IDPs, α- and β-casein. We found that with respect to their effect on IDPs, osmolytes can serve as either folding inducers or folding evaders. Folding evaders do not have any structural and functional effects on IDPs, while folding inducers such as taurine and TMAO^29^ do alter IDP structure and impede their function. The study hints that unwanted production and/or accumulation of certain osmolytes at high levels may alter structure and function of certain IDPs and therefore underlie pathophysiology of various diseases.

## Results

It is well established that α- and β-caseins exhibit chaperone activity, wherein they have been shown to suppress thermally- and chemically-induced aggregation of various substrate proteins. Therefore, we investigated the effect of various osmolytes on the chaperone activity of α-casein by measuring the aggregation kinetics of catalase at 55 °C. Catalase at this temperature has been found to form amorphous aggregates. At least two osmolytes were chosen from each class, with the exception for methylamines as their effect on the same protein system has been already published from our laboratory^29^.

**Figure 1:**
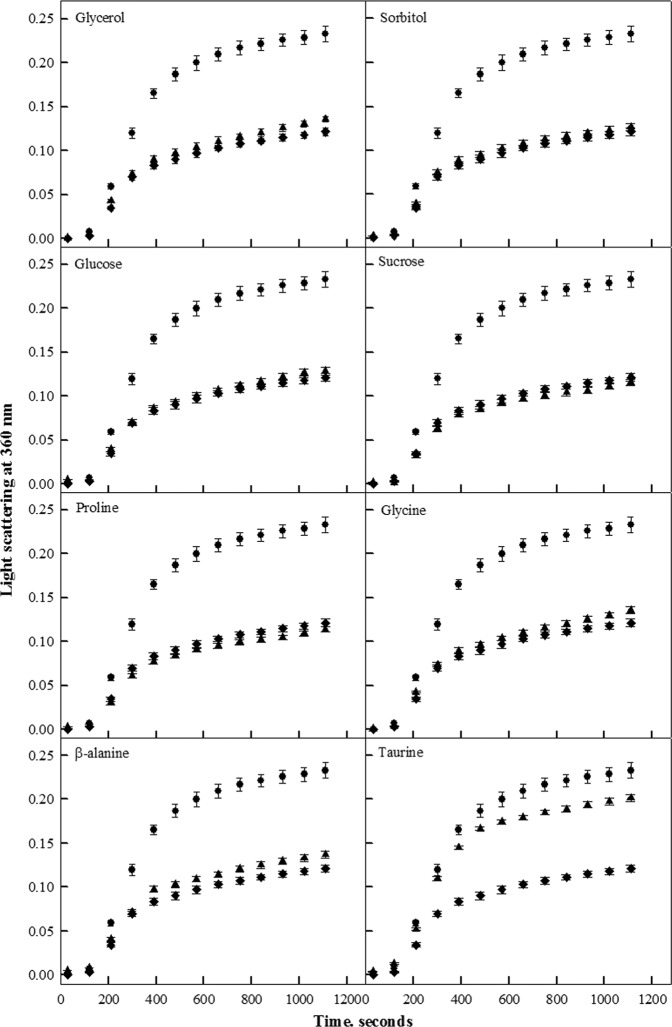
Effect of osmolytes on the chaperone activity of α-casein: Chaperone activity of α-casein was monitored by measuring light scattering intensity of catalase at 360 nm in presence of glycerol, sorbitol, glucose, sucrose, proline, glycine, β-alanine and taurine. Symbols denote (**— —**) catalase control, (**—•—**) catalase in presence of untreated-α-casein and (**—••—**) catalase in presence of osmolyte treated-α-casein. For clarity we have shown aggregation profiles of catalase in presence of only the highest concentration (500 mM) of the osmolyte used. Results shown are representative of atleast three independent measurements for all the osmolytes.

The aggregation kinetic profiles of catalase obtained in the absence and presence of various osmolytes are shown in Fig. 1. Although a wide range of the concentrations were used for all the osmolytes under study, for clarity we have only shown the profiles obtained at the highest concentration, i.e., 500 mM of the osmolytes used. Percent change in the extent of catalase aggregation in the presence of osmolyte-treated α-casein is given in Table 1. It is seen in Fig. 1 that as compared to catalase alone, α-casein inhibits heat-induced aggregation of catalase as revealed by the decreased light scattering intensity at 360 nm. This eventually led to about 48% decrease in the extent of catalase aggregation (Table 1). Interestingly, Fig. 1 shows that, except for taurine, the aggregation profiles of catalase in the presence of osmolyte-treated α-casein are similar to those obtained with the untreated α-casein, indicating that these osmolytes do not significantly alter the chaperone activity of α-casein. On the other hand, taurine was found to reduce the α-casein chaperone activity as revealed by increased light scattering intensity of aggregating catalase in the presence of taurine-treated α-casein as compared to the untreated α-casein (Fig. 1h). In fact, in presence of highest concentration of taurine used in this study, only a meagre 14% decrease in the extent of catalase aggregation was observed.

**Table 1:** Effect of osmolytes on the extent of decrease in catalase aggregation in presence of osmolyte treated and untreated α-casein.

Name of osmolyte	% decrease in extent of Catalase aggregation^*^					
	α-casein alone	α-casein with 50 mM	α-casein with 100 mM	α-casein with 250 mM	α-casein with 500 mM	Osmolyte alone^**^
Glycerol	48	44	43	41	41	04
Sorbitol	48	45	44	43	43	03
Glucose	48	44	43	42	42	02
Sucrose	48	49	50	50	51	13
Glycine	48	43	41	41	40	0
Proline	48	50	52	53	54	11
β-alanine	48	42	41	40	40	03
Taurine	48	41	30	24	14	22

^*^Error observed from triplicate measurements were in the range of 3–5%. **The measurements in case of osmolyte alone were carried out at highest concentration only.

**Figure 2:**
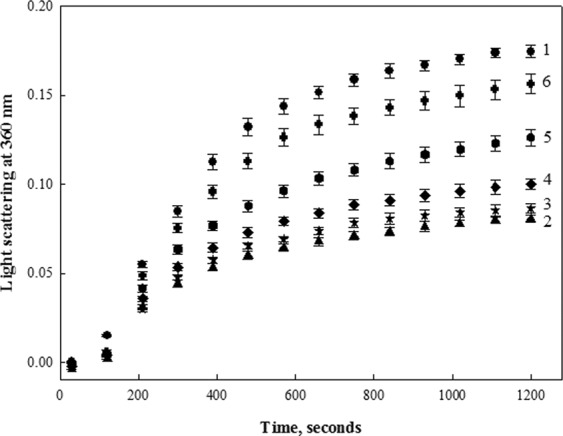
Effect of taurine on the chaperone activity of β-casein: Chaperone activity of β-casein in presence of taurine was monitored by measuring light scattering intensity of catalase at 360 nm in presence of different concentrations of taurine. Curve 1 (catalase control), curve 2 (catalase in presence of untreated- β-casein alone), curve 3 (catalase in presence of 50 mM taurine treated- β-casein), curve 4 (catalase in presence of 100 mM taurine treated- β-casein), curve 5 (catalase in presence of 250 mM taurine treated-β-casein), curve 6 (catalase in presence of 500 mM taurine treated-β-casein). Results shown are representative of atleast three independent measurements for all the osmolytes.

Furthermore, using similar experimental procedures, the effect of taurine on another IDP, β-casein, was also investigated. It was observed that similar to α-casein, taurine inhibited the chaperone activity of β-casein (Fig. 2). In order to see whether osmolyte alone could also influence the aggregation of catalase, effect of osmolytes (highest concentration) on the aggregation of catalase was monitored by measuring light scattering intensity at 360 nm for 20 minutes. From these experiments, it was observed that none of the osmolyte used in the study was able to noticeably accelerate the aggregation of catalase, whereas taurine, proline and sucrose showed some minor propensity to decrease catalase aggregation.

**Figure 3:**
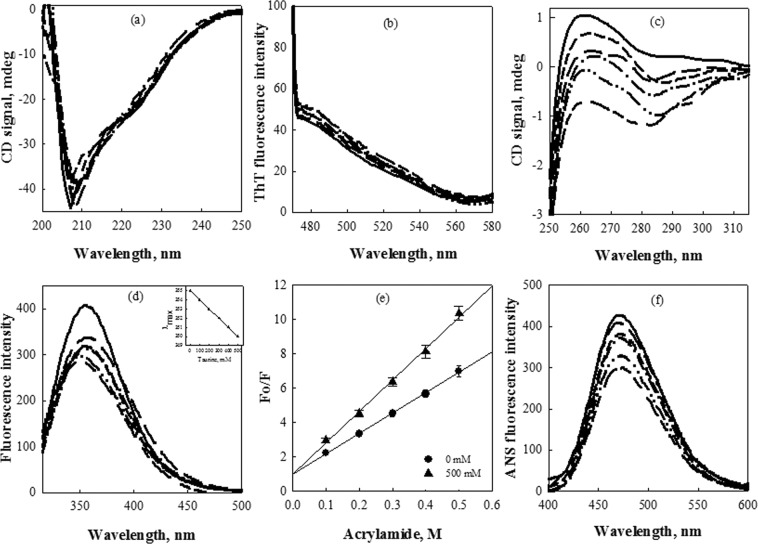
Effect of taurine on the secondary and tertiary structure of α-casein: Far- UV CD spectra (**a**), ThT fluorescence intensity (**b**), near-UV CD spectra (**c**), intrinsic tryptophan fluorescence (**d**), acrylamide quenching (**e**) and ANS fluorescence intensity (**f**) of α-casein in presence of different concentrations of taurine 0 mM (**——)**, 50 mM (**— —)**, 100 mM (**– – –)**, 250 mM (**—•—)**, 350 mM (**—••—)**, 500 mM (**— – –**). Acrylamide quenching has been performed only at highest concentration of taurine. Spectra shown are representative of atleast three independent measurements with error rates as mentioned in Table 2.

**Figure 4:**
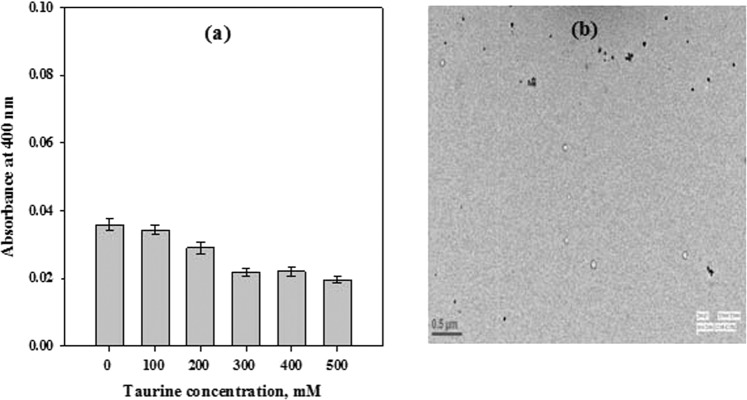
Light scattering and TEM of α-casein in presence of taurine: Measurement of light scattering at 400 nm by α-casein in presence of different concentrations of taurine (**a**), TEM image of α-casein in the presence of 500 mM taurine. (**b**) The scale bar is 0.5 µm. Results shown are mean of atleast three independent measurements with error bars as the standard error of the mean.

To further investigate if taurine has any effect on the structural properties of α-casein, alterations in structure of α-casein were investigated in presence of taurine (Fig. 3). This structural analysis revealed that even at highest concentration (Fig. 3a), no significant change in the negative ellipticity around 222 nm and 204 nm of far- UV circular dichroism (CD) spectra was observed, indicating that taurine has minimal effect on the mostly disordered secondary structure of α-casein. As can be seen in Fig. 3b, the absence of ThT binding further revealed that no transition of the conformation towards β-sheet or cross β-sheet formation has occurred. However, a taurine concentration dependent increase in the negative ellipticity of α-casein in the near-UV CD spectra (wavelength range of 275–290 nm) was observed (Fig. 3c), suggesting formation of new tertiary contacts or some structural compaction. Similarly, tryptophan fluorescence measurements of α-casein in presence of taurine showed a taurine concentration-dependent decrease in the fluorescence intensity (Fig. 3d) accompanied with a prominent blue shift (inset in Fig. 3d). These observations suggest that in the presence of taurine, the environment of tryptophan residues of α-casein became more hydrophobic, indicating some compaction of this protein leading to the burial of its aromatic residues. Acrylamide quenching of α-casein intrinsic tryptophan fluorescence was also investigated by measuring the acrylamide-induced change in the intensity of the tryptophan emission spectra in presence of taurine (Fig. 3e). It was observed that there was a noticeable increase in the efficiency of acrylamide quenching upon treatment with taurine, clearly indicating changes in the accessibility of tryptophan residues to solvent and quencher. ANS (1-anilinonapthalene-8-sulfonic acid) binding studies further indicated that as compared to untreated α-casein, a decreased binding of ANS to taurine treated α-casein was observed (Fig. 3f). In order to rule out any taurine-induced aggregation of α-casein, light scattering intensity and transmission electron microscopy (TEM) measurements were carried out with α-casein in the presence of different concentrations of taurine. As seen in Fig. 4, there was no significant increase in the light scattering intensity of α-casein upon treatment with taurine (Fig. 4a), and also there was no visible aggregate formation as revealed by TEM (Fig. 4b). Altogether, these results show complete absence of any casein aggregates in presence of taurine.

**Figure 5:**
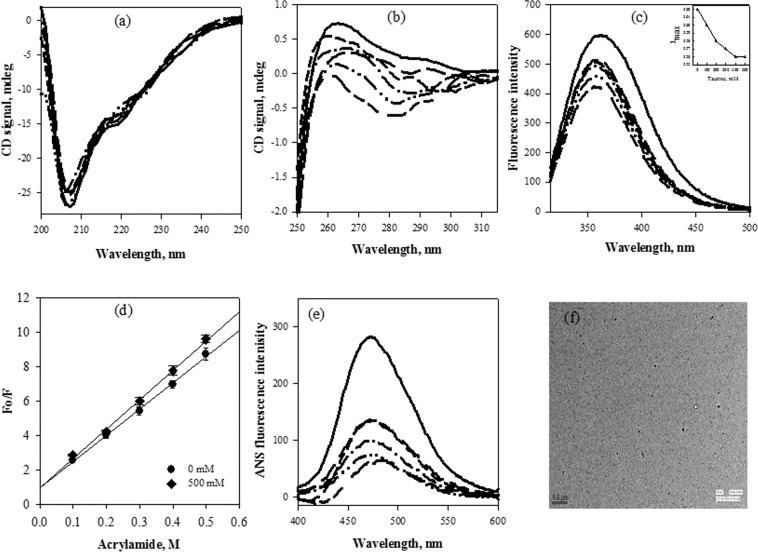
Effect of taurine on the conformation of β-casein: Far- UV CD spectra (**a**), near-UV CD spectra (**b**), intrinsic tryptophan fluorescence (**c**), acrylamide quenching (**d**), ANS fluorescence intensity (**e**) and TEM (**f**) of β-casein in presence of different concentrations of taurine 0 mM (**——**), 50 mM (**— —**), 100 mM (**– – –**), 250 mM (**—•—**),350 mM (**—••—**), 500 mM (— – –). Acrylamide quenching has been performed only at 500 mM taurine. Spectra shown are representative of atleast three independent measurements with error rates as mentioned in Table 2.

Similar experiments were also conducted for monitoring the effect of taurine on β-casein structure (Fig. 5). It can be seen in Fig. 5a that similar to α-casein, there was no change in the secondary structure of β-casein as revealed by the lack of taurine-induced changes in the far-UV CD spectra of β-casein. On the other hand, taurine promoted a noticeable increase in the tertiary structure of β-casein, as shown by changes in near-UV CD spectra (Fig. 5b). Furthermore, results of the analysis of the taurine effects on intrinsic fluorescence and efficiency of acrylamide quenching of intrinsic fluorescence of β-casein were similar to those reported for α-casein (Fig. 5c,d and inset in 5c). A significant decrease in the ANS fluorescence intensity was observed in the presence of taurine relative to that of the untreated β-casein. This decreased ANS binding in turn suggests decreased exposure of hydrophobic patches of the IDP in presence of taurine (Fig. 5e). Additionally, no aggregate formation was observed upon treatment of β-casein by taurine (Fig. 5f).

Structural analyses of these IDPs were also carried out in the presence of the other osmolytes (glycerol, sorbitol, glucose, sucrose, proline, glycine, and β-alanine). All these osmolytes were found to have insignificant effect on the overall structure of α-casein (see Supplementary Fig. S1). None of the osmolytes had any significant effect on the secondary structure of α-casein. Tryptophan fluorescence studies also revealed no significant change in the emission spectra of α-casein in the presence of any of the osmolytes (see Supplementary Fig. S2). In order to look for any change in the exposure of hydrophobic groups of α-casein in presence of the osmolytes, ANS binding assays were performed. It was observed that none of the osmolytes had any effect on the exposure pattern of hydrophobic groups to the solvent as no significant change in the ANS fluorescence intensity was observed in osmolyte-treated α-casein relative to the untreated α-casein (see Supplementary Fig. S3).

## Discussion

**Figure 6:**
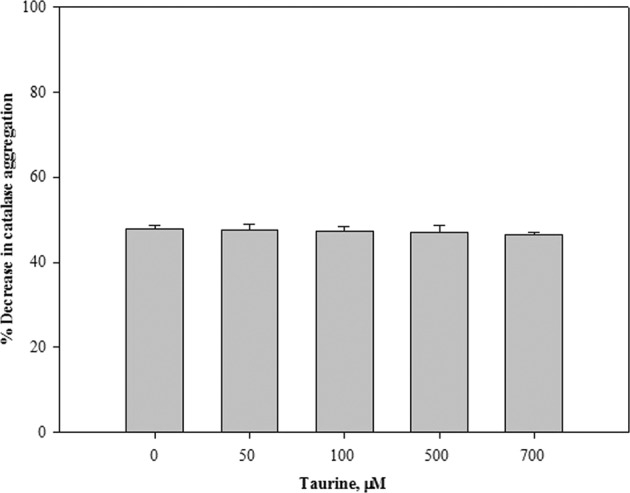
Effect of taurine at physiological concentration range on the chaperone activity of α-casein: For this, percent decrease in catalase aggregation was calculated in presence of different physiological concentrations of taurine. Results shown are mean of three independent measurements with error bars as the standard error of the mean.

To examine the possibility that osmolytes can regulate functions of IDPs, different osmolytes, representing each chemical class (polyols, sugars, amino acids and their derivatives, methylamines),were screened for their effect on the chaperone activity of α-casein (Fig. 1 and Table 1). It was observed that except taurine, all other osmolytes have no significant effect on the chaperone activity of α-casein. The observed effect of osmolytes, other than taurine, on chaperone activity of α-casein is quite consistent with the results obtained for some other IDPs^29,38^. Being known as compatible solutes, the osmolytes are expected to not affect the functioning of IDPs as it was observed earlier for some folded globular proteins^39–41^. However, surprisingly, in our case, taurine decreased chaperone activity of α-casein in a concentration dependent manner (Fig. 1h). In fact, the inhibitory effect was not found to be confined to α-casein only, as the chaperone activity of β-casein was also decreased by taurine (Fig. 2). Interestingly, taurine could not completely inhibit chaperone activity of α-casein as the aggregation of catalase goes up to 86% in the presence of taurine-treated α-casein. This meagre 14% decrease in the extent of catalase aggregation by the taurine treated α-casein as compared to the 48% decrease in α-casein alone is quite surprising. It is worth noting that taurine alone can decrease the heat-induced aggregation of catalase by 22% (Table 1).Therefore, it might be possible that taurine alters α-casein chaperone activity and at the same time inhibits heat-induced aggregation of catalase. Both α-casein and taurine have been found to co-exist in the mammalian breast milk, with taurine concentration ranging from 100–700 μM^42,43^. However, during stress and disease conditions, concentration of osmolytes may go up to millimolar range^29,37^. We have further investigated if the physiological concentration of taurine could also alter the α-casein chaperone activity. It was observed that at physiological concentrations, taurine has no significant effect on the chaperone activity of α-casein (Fig. 6). Results obtained in the present study do infer that taurine at concentrations beyond the physiological range may alter the α-casein chaperone activity.

It is quite possible that taurine and casein proteins might have naturally co-evolved in such a way that the co-existence of taurine and casein proteins is regulated in a mutual fashion, so as to maintain the structural and functional plasticity of these IDPs. Interestingly, osmolytes have been found to buffer mutational defects in proteins^44^. Taurine is not only an osmolyte but also serves as antioxidant and is important for the development and function of skeletal muscles, retina, central nervous system, and regulation of cardiovascular function^45–50^. It has been reported that corpora amylacea, observed in alveoli lined with active secreting cells of bovine mammary gland, may be caused by the aggregation, fusion, and compaction of casein proteins^51^. Results obtained in this study do indicate that taurine may be involved in the disease pathophysiology as the decreased chaperone activity of the casein proteins in presence of taurine may led to aggregation of various milk proteins, resulting in protein aggregation. It is worth mentioning that casein proteins under normal conditions prevent aggregation of milk proteins^52–54^.

It is well known that osmolytes can induce folding of naturally foldable proteins into compact three dimensional structures under the denaturing conditions^55,56^. This ability of osmolytes to protect proteins against denaturing stresses is predominantly due to their preferential exclusion from the protein surface originating from their unfavourable interactions with the protein peptide backbone^57,58^. This tendency of osmolytes to be excluded from proteins forces them to acquire conformations with least exposed surface areas. In light of this, the loss of chaperone activity of casein proteins in the presence of taurine might be either due to (i) structural alterations, (ii) partial folding of the IDPs leading to the formation of a compact structure, or (iii) aggregate formation as compaction due to the preferential hydration effect of taurine on casein proteins might promote protein aggregation, which is one of the major outputs of the compaction of IDPs.

These possibilities prompted us to investigate the structural consequences of the presence of taurine on α-casein. As evident from Fig. 3, although there is no significant effect of taurine on protein secondary structure (Fig. 3a),the tertiary structure content in α-casein does increase in the presence of taurine (Fig. 3c). Mostly unaltered secondary structure with creation of significant tertiary structure indicates that taurine might induce partial folding of the intrinsically disordered α-casein. For this to happen, burial of aromatic and hydrophobic groups to the core of the newly created structure is expected. To probe for the effect of taurine on the environment of aromatic groups, intrinsic tryptophan fluorescence and acrylamide quenching were analysed. A concentration dependent decrease in tryptophan fluorescence accompanied with the prominent blue shift revealed shifting of tryptophan residues to a relatively non-polar environment (Fig. 3d). One possible reason for the reduction in fluorescence intensity is intramolecular quenching by disulphides, phenylalanine, and histidine amino acids, which might have come in close proximity to tryptophan upon structural collapse. Acrylamide quenching further confirmed that, as compared to untreated α-casein, an increased quenching in case of taurine-treated α-casein indicates that taurine induced compaction might have brought inaccessible tryptophan residue (s) in close proximity to various quenchers (Fig. 3e). To monitor the exposure pattern of hydrophobic patches, ANS binding assays were performed. ANS is an extrinsic fluorophore which fluoresces strongly upon binding to an exposed hydrophobic patch and shows least fluorescence in case of fully folded globular proteins with hydrophobic amino acid patches buried inside the core of protein^59,60^. With increasing concentration of taurine, a decrease in ANS binding was observed for α-casein suggesting burial of most of the hydrophobic groups that were exposed in case of untreated α-casein (Fig. 3f). Similar structural consequences were observed in the case of β-casein as well (Fig. 5). Taken together, all these results do confirm that taurine induces partial folding of casein proteins leading to the creation of additional tertiary interactions.

Recently we have been able to show that similar to taurine, as observed in this study, TMAO also induces partial folding of α-casein^29^. It has been observed that due to the presence of less number of hydrophobic residues and high number of charged amino acids, IDPs do not fold to a globular conformation under the normal physiological conditions. However, the TMAO-induced partial hydrophobic collapse of α-casein driven by clustering of its hydrophobic residues increases the chances of protein aggregation due to the interaction of exposed hydrophobic clusters with each other. In order to rule out this possibility, we have examined if the taurine-induced collapsed structure of casein undergoes aggregate formation using light scattering and TEM. Interestingly, no aggregate formation was observed (Fig. 4).

Altogether, results reported in this study indicate that the taurine-collapsed forms of α- and β-casein are although quite stable but have an altered chaperone activity. It is generally believed that exposed hydrophobic patches of amino acids intercepted by hydrophilic amino acid groups are essential for chaperone function of IDPs^61^. In conformity to this, the burial of the hydrophobic patches of α-casein in presence of taurine might be responsible for the loss of its chaperone activity. Enhanced inaccessibility of hydrophobic patches in taurine-treated casein may decrease their binding to the aggregating substrate proteins and thus making the casein proteins incapable for preventing aggregation of catalase.

**Figure 7:**
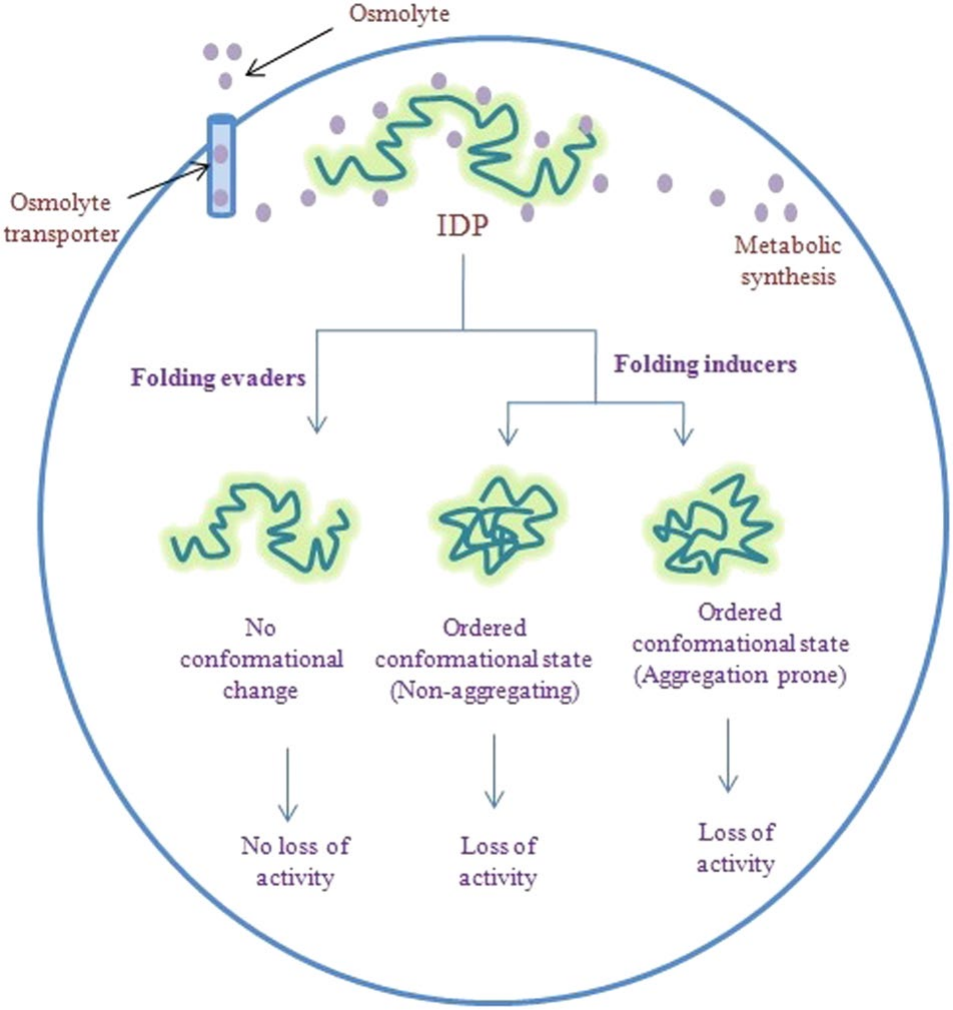
Schematic representation of possible consequences of osmolyte-induced structural alterations in IDPs on their chaperone activity.

We then investigated the effect of other osmolytes on the structure of α-casein. As can be seen in Supplementary Fig. S1, similar to taurine, all other osmolytes do not have any significant effect on the secondary structure of α-casein. However, in contrast to taurine that induces tertiary structural changes, all other osmolytes do not influence the tertiary structure of α-casein (see Supplementary Figs. S2 and S3). Taken together, these results indicate that taurine induces partial collapse of α-casein while other osmolytes could not do so. It appears that the unfavourable interactions offered by taurine with α-casein are overwhelmingly large enough to induce folding as compared to that of the other osmolytes. Based on the present study and other studies reported earlier^29,38^ different osmolytes may have different consequences with respect to structural integrity of IDPs that might have led to the diverse effects on the chaperone activity of casein proteins (Table 2). One group of osmolytes (glycerol, sorbitol, glucose, sucrose, proline, glycine, and β-alanine) do not affect the structural disorderness (due to insignificant effects on secondary and tertiary structures) leading to unaltered chaperone activity of casein proteins. Other group of osmolytes exhibited no significant alteration (taurine) to slight increase (TMAO) in the secondary structure but both induced partial folding of the α-casein leading to formation of new tertiary interactions as evident by increase in tryptophan fluorescence intensity (Table 2). A subtle difference in the structure of the osmolyte induced collapsed structure of the IDPs, marked by exposure of hydrophobic cluster as evidenced by increased ANS binding to the solvent, leads to the casein aggregate formation in case of TMAO (Table 2) while in case of taurine the burial of hydrophobic cluster to the core (as evidenced by decrease in ANS binding) leads to loss of chaperone activity of casein proteins with no casein aggregate formation. The differences in the effect of taurine and TMAO on IDPs might be due to their different effects on the water structure in the immediate surroundings of the proteins. Figure 7 summarizes the effect of osmolytes on IDPs wherein it has been shown that based on their effect on casein proteins, osmolytes can act as either folding inducers or folding evaders. Folding evaders include those osmolytes that are not able to induce folding of IDPs and hence no functional alterations while folding inducers may induce folding that can either lead to aggregation of IDPs or fold inappropriately in such a way that it fails to bind substrates. Thus, attempts to fold IDPs may have unusual functional consequences.

**Table 2:** Effect of osmolytes on the structural and functional parameters of α-casein.

Osmolyte	Structural and Functional parameters^#^				
	Δθ_222_	%Δ_fl_	%Δ_ANS_	Absorbance at 400 nm^*^	% Decrease in catalase aggregation**
Taurine	−(0.85)	−(31)	−(29.4)	0.012	14
TMAO	2.7	−(17.6)	19.7	0.165	05
Glycerol	−(0.24)	−(04.0)	−(0.90)	0.018	41
Sorbitol	0.35	−(06.2)	01.8	0.012	43
Glucose	−(0.39)	−(04.4)	01.3	0.015	42
Sucrose	01	−(08.0)	07.5	0.011	51
Glycine	0.0	−(12.7)	01.0	0.011	40
Proline	—	−(05.9)	0.70	0.011	54
β-alanine	−(0.35)	−(04.4)	01.7	0.010	40
Sarcosine	0.08	−(04.4)	07.2	0.016	41
Betaine	−(0.1)	−(06.3)	02.7	0.017	49

*Absorbance of α-casein alone was observed to be 0.019, ^**^α-casein alone inhibits catalase aggregation by 48%. ^#^Δθ_222_ is change in ellipticity at 222 nm of Far- UV CD spectra, %Δ_fl_ is the percent change in tryptophan fluorescence intensity, %Δ_ANS_ is the percent change in ANS fluorescence intensity. From the independent measurements, the maximum error from the mean are in the range of 3–5% in θ_222_, 5–7% in tryptophan fluorescence, 5–8% in ANS assay, and 3–8% in case of absorbance studies.

In summary, many osmolytes in general do not induce folding of IDPs and therefore are compatible with their structural and functional integrity. However, certain osmolytes may induce structural collapse of IDPs and will eventually lead to altered functional activity of these IDPs. Both the folding inducers i.e., taurine and TMAO have been identified to be associated with various clinical complications including cardiovascular, neurodegeneration, atherosclerosis and cancer^62–66^. Interestingly, it has been recently proposed that osmolyte induced alterations in the transcription factors are sufficient for establishing some novel protein signalling networks that may favour metastasis^67^. It is worth noting that ability of transcription factors to interact with multiple ligands during various cellular processes is due to their intrinsically disordered domains. Our study hints towards the involvement of excessive accumulation of taurine or TMAO in pathophysiology of various diseases and thus could be used as biomarkers for such diseases^68,69^. These findings open up a new avenue wherein unwanted expression or enhanced accumulation of osmolytes like taurine or TMAO might be a risk factor for a large number of human diseases as the functional failure of IDPs by them may affect important signalling pathways.

## Experimental Procedure

### Materials

Lyophilized protein Bovine α- and β-casein, glycerol, sorbitol, glucose, sucrose, taurine, β-alanine, sodium cacodylate trihydrate, acrylamide, ThT, ANS were purchased from Sigma-Aldrich Chemical Company USA. Potassium chloride and sodium hydroxide were purchased from Merck India. No further purification of chemicals of analytical grade was done before usage. Both α- and β-casein were dialyzed at 4 °C extensively against KCl (0.1 M, pH 7.4). Syringe filters with 0.22 µm pore size were used to filter stock solutions of proteins. Cary-300 UV/Visible spectrophotometer was used to determine the protein concentrations of α- and β-casein using molar absorbance coefficient (ε) values of 11,000 M − ^1^cm^−1^ and 11,460 M − ^1^cm^−^ respectively at 280 nm. Degassed cacodylate buffer (0.05 M, pH 7.4) was used in all the measurements.

### Activity measurements

Chaperone activity of α- and β-casein was determined against catalase, which amorphously aggregates at 55 °C, in absence and presence of different concentrations of taurine (50, 100, 250 and 500 mM). For this, light scattering measurement at 360 nm for 20 minutes was carried out using Cary-300UV/Visible spectrophotometer equipped with a peltier-type temperature controller. Catalase, with a concentration of 0.38 mg/ml, was taken in 1:1 ratio with α- and β-casein.

### Circular dichroism (CD) measurements

Far- and Near- UV CD measurements of α- and β-casein in absence and presence of different concentrations of the osmolytes were carried out on Jasco J-810 spectropolarimeter. Quartz cuvettes of 0.1 and 1.0 cm path length were used for Far- (200–250 nm) and Near- (240–320 nm) UV CD respectively. All the measurements were recorded in triplicates. Protein concentrations of 0.35 mg/ml and 0.50 mg/ml were used for Far- and Near- UV CD measurements, respectively. The scan rate used was 100 nm/min with a band width of 1 nm and 5 nm for Far- and Near-UV CD respectively.

### Fluorescence measurements

Fluorescence spectra were collected in a Perkin Elmer LS 55 spectrofluorimeter using a 3 mm quartz cuvette. The excitation wavelength was set to 295 nm and the emission spectra were collected from 300–500. Slit widths for both excitation and emission were set to 10 nm with a scanning rate of 100 nm/min. at 25 °C. Both proteins were used at a concentration of 3 μM. All the measurements were recorded in triplicates.

Extrinsic fluorescence measurements were performed using ANS dye with excitation wavelength of 350 nm and the emission spectra were collected from 400–600 nm.ANS concentration was taken 16 times higher than that of protein concentration. ANS was added to the samples 30 minutes prior to collecting spectra. Each spectrum was corrected for appropriate buffer contribution and was done in triplicates.

ThT fluorescence measurements of α- and β-casein were performed using excitation wavelength of 442 nm and emission spectra were observed from 460–600 nm. ThT concentration of 5 μM was used for the assay. Proteins were incubated with ThT for 30 minutes before collecting spectra. All the experiments were done in triplicates and each spectrum was corrected for appropriate buffer contributions.

### Transmission electron microscopy

Samples of α- and β-casein, in absence and presence of 500 mM taurine, were placed on a copper grid (100 mesh * 250 µm pitch) and incubated at room temperature for 5 minutes. Negative staining of the samples present on copper grids was done using uranyl acetate solution (1.0%). After air drying, samples were examined using FEI Tecnai G2-200kV HRTA transmission electron microscope operating at 200 kV.

### Light scattering measurements

In order to look for any taurine induced aggregation of casein proteins, light scattering measurements were carried out at 400 nm using Cary-300 UV/Visible spectrophotometer. α- and β-casein were taken at a concentration of 0.5 mg/ml. All the measurements were recorded in triplicates.

## Supplementary information

Supplementary Figures
