# Supplementary material for: Taurine Induces an Ordered but Functionally Inactive Conformation in Intrinsically Disordered Casein Proteins
Source: Sci Rep. 2020 Feb 26;10:3503. doi: 10.1038/s41598-020-60430-7 (PMC7044306; doi:10.1038/s41598-020-60430-7)
Supplement: Supplementary file 1 — Supplementary Figures [file 41598_2020_60430_MOESM1_ESM.pdf]

Taurine Induces an Ordered but Functionally Inactive Conformation in Intrinsically Disordered Casein Proteins

Mohd Younus Bhat[1], Laishram Rajendrakumar Singh[2*], Tanveer Ali Dar[1*]

[1]Department of Clinical Biochemistry, University of Kashmir, Srinagar, J&K, 190006, India. [2]Dr.B. R. Ambedkar Center for Biomedical Research, University of Delhi, Delhi, 110007, India.

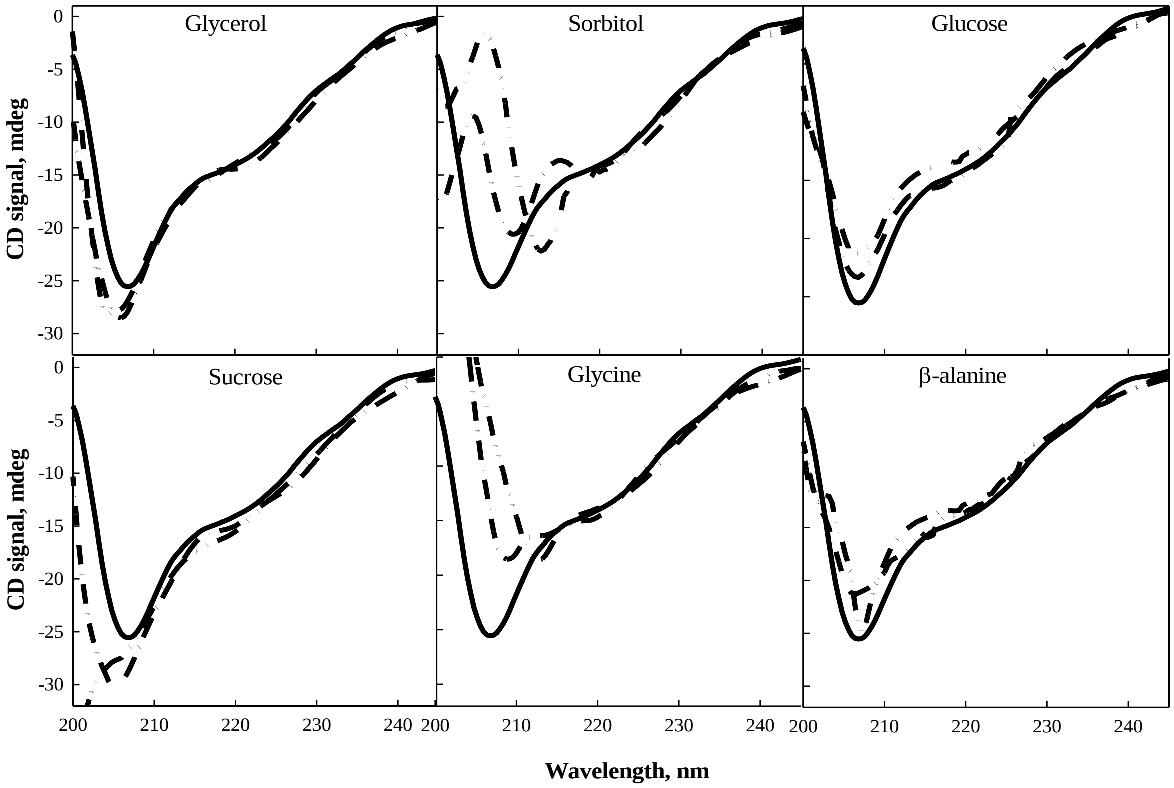

Figure S1: Effect of osmolytes on Secondary structure of α-casein in presence: Far- UV CD spectra of α-casein was measured in presence of different osmolytes. Far- UV CD spectra of α-casein in presence of proline could not be measured due to high voltage issues. For clarity, we have shown only the far UV CD spectra of α-casein in presence of 0 mM (——), 250 mM (—•—) and 500 mM(—••—) of the osmolyte only. Spectra shown are representative of atleast three independent measurements with average error in the range of ± 0.5.

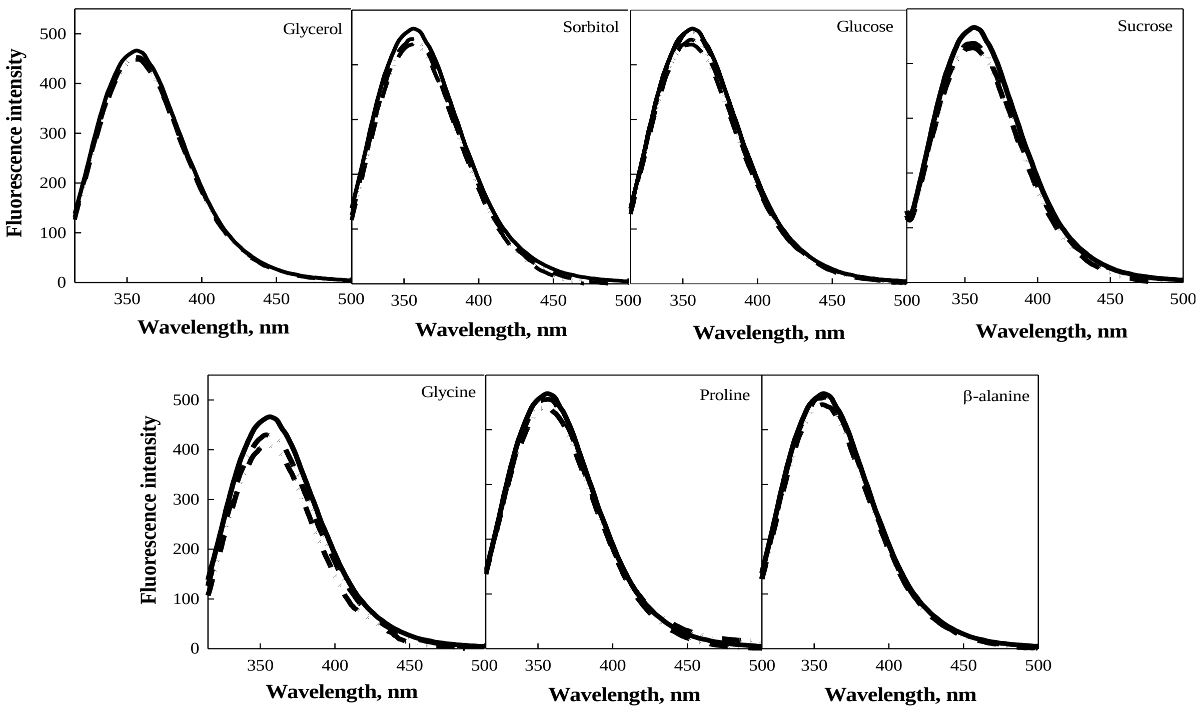

Figure S2: Intrinsic fluorescence measurement of α-casein in presence of osmolytes: Intrinsic tryptophan fluorescence of α-casein was measured in presence of different osmolytes. For clarity, we have shown only the spectra for 0 mM (——), 250 mM (—•—)and 500 mM(—••—) of the osmolyte. Spectra shown are representative of atleast three independent measurements with an average error in the range of ± 5.

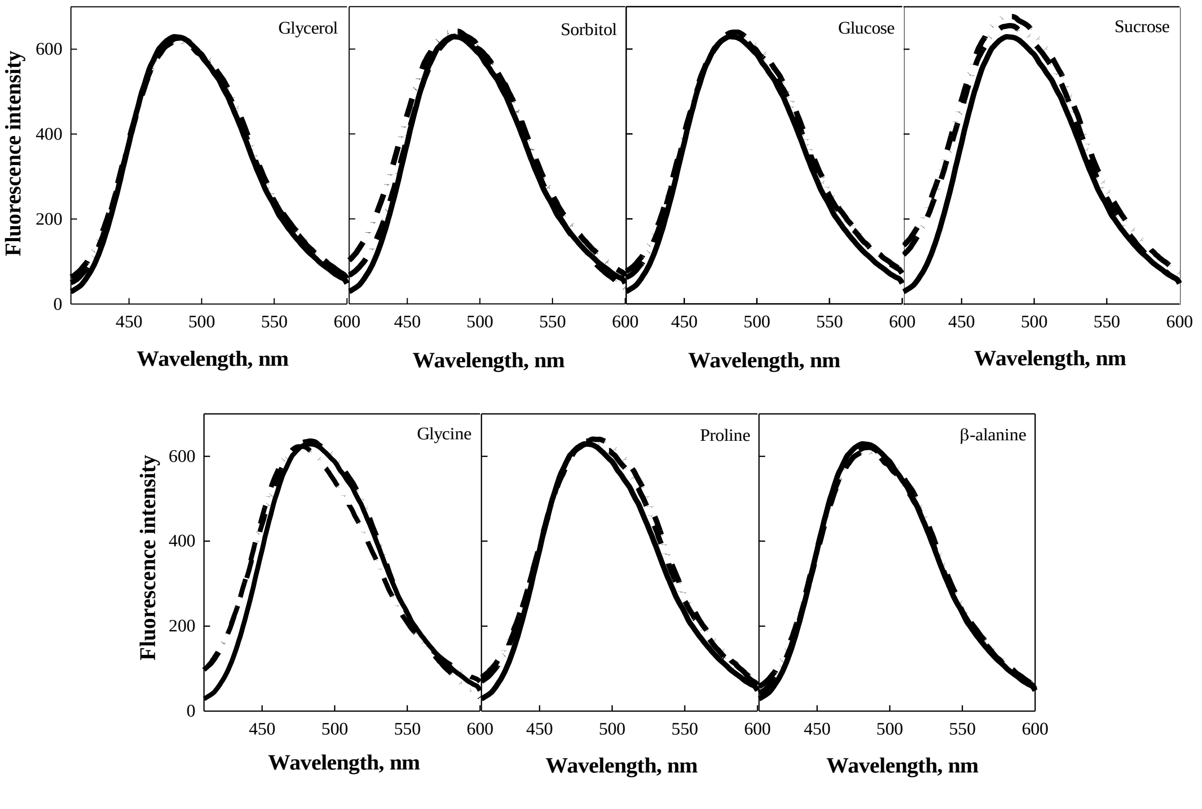

FIGURE S3: Extrinsic ANS fluorescence measurement of α-casein in presence of osmolytes: ANS fluorescence spectra of α-casein was performed in presence of different osmolytes. For clarity, we have shown only the spectra for 0 mM (——), 250 mM (—•—)and 500 mM (—••—) of the

osmolyte. Spectra shown are representative of atleast three independent measurements with an average error in the range of ± 7.
